# A 5-Year Follow-up of Internet-Based Cognitive Behavior Therapy for Social Anxiety Disorder

**DOI:** 10.2196/jmir.1776

**Published:** 2011-06-15

**Authors:** Erik Hedman, Tomas Furmark, Per Carlbring, Brjánn Ljótsson, Christian Rück, Nils Lindefors, Gerhard Andersson

**Affiliations:** ^5^Department of Behavioural Sciences and LearningLinköping UniversityLinköpingSweden; ^4^Swedish Institute for Disability ResearchLinköping UniversityLinköpingSweden; ^3^Department of PsychologyUmeå UniversityUmeåSweden; ^2^Department of PsychologyUppsala UniversityUppsalaSweden; ^1^Department of Clinical NeuroscienceKarolinska InstitutetStockholmSweden

**Keywords:** Internet, cognitive behavior therapy, anxiety disorders, social anxiety disorder, 5-year follow-up

## Abstract

**Background:**

Internet-based cognitive behavior therapy (CBT) has been shown to be a promising method to disseminate cognitive behavior therapy for social anxiety disorder (SAD). Several trials have demonstrated that Internet-based CBT can be effective for SAD in the shorter term. However, the long-term effects of Internet-based CBT for SAD are less well known.

**Objective:**

Our objective was to investigate the effect of Internet-based CBT for SAD 5 years after completed treatment.

**Method:**

We conducted a 5-year follow-up study of 80 persons with SAD who had undergone Internet-based CBT. The assessment comprised a diagnostic interview and self-report questionnaires. The main outcome measure was the Liebowitz Social Anxiety Scale-Self-Report (LSAS-SR). Additional measures of social anxiety were the Social Interaction Anxiety Scale (SIAS) and the Social Phobia Scale (SPS). Attrition rates were low: 89% (71/80) of the participants completed the diagnostic interview and 80% (64/80) responded to the questionnaires.

**Results:**

Mixed-effect models analysis showed a significant effect of time on the three social anxiety measures, LSAS-SR, SIAS, and SPS (*F*_3,98__-102_ = 16.05 - 29.20, *P* < .001) indicating improvement. From baseline to 5-year follow-up, participants’ mean scores on the LSAS-SR were reduced from 71.3 (95% confidence interval [CI] 66.1-76.5) to 40.3 (95% CI 35.2 - 45.3). The effect sizes of the LSAS-SR were large (Cohen’s *d* range 1.30 - 1.40, 95% CI 0.77 - 1.90). Improvements gained at the 1-year follow-up were sustained 5 years after completed treatment.

**Conclusions:**

Internet-based CBT for SAD is a treatment that can result in large and enduring effects.

**Trial registration:**

Clinicaltrials.gov NCT01145690; http://clinicaltrials.gov/ct2/show/NCT01145690 (Archived by WebCite at http://www.webcitation.org/5ygRxDLfK)

## Introduction

Social anxiety disorder (SAD) is common [1], is associated with functional impairment [2], and often becomes chronic if left untreated [3]. In recent years, Internet-based cognitive behavior therapy (CBT) has demonstrated efficacy in several randomized controlled trials [4-9]. In general, effect sizes on measures of social anxiety in these studies have been at parity with those seen in trials investigating conventional CBT (Cohen’s *d* typically ranging from 1.0-1.5) [10,11]. In essence, Internet-based CBT could be described as Internet-administered self-help therapy with online therapist contact and support. The treatment components and theoretical basis are the same as in conventional CBT. While several studies have shown that conventional CBT produces long-term improvements up to 5 years after treatment [12-15], nearly all studies on Internet-based CBT have had a follow-up period of 1 year or shorter. The one exception is a study where participants receiving Internet-based CBT not only maintained their treatment gains but also were further improved at a 2.5-year follow-up [16]. This is in line with the notion that reduced anxiety following CBT to a large extent is contingent on repeated exposure [17].

The aim of the present study was to investigate the effects of Internet-based CBT for SAD 5 years after treatment, as no previous study has investigated if the effect of Internet-based CBT persists over this long period of time. We hypothesized that treatment gains would be sustained on measures of social anxiety, depressive symptoms, general anxiety, and quality of life.

## Methods

### Design

This was a follow-up study assessing 80 participants who had received Internet-based CBT for SAD within the context of a randomized controlled trial (RCT) conducted in 2005. In the original RCT, participants were randomized to treatment (n = 40) or waiting list control (n = 40) with equal probability. Participants were randomized using a true random number service (http://www.random.org). Participants were randomized after inclusion in the study, ensuring that allocation status was unknown to the assessors deciding on inclusion. Following treatment and postassessment, participants in the waiting list control group were crossed over to treatment. Thus, both groups had received Internet-based CBT at 1-year follow-up. As the two groups received treatment at different time points, results are reported separately for the two groups. CBT denotes the first group, and waiting list (WL)-CBT, the latter. A detailed description of the original study is available elsewhere [6]. The trial was registered at clinicaltrials.gov (identifier NCT01145690).

### Sample and Recruitment

**Figure 1:**
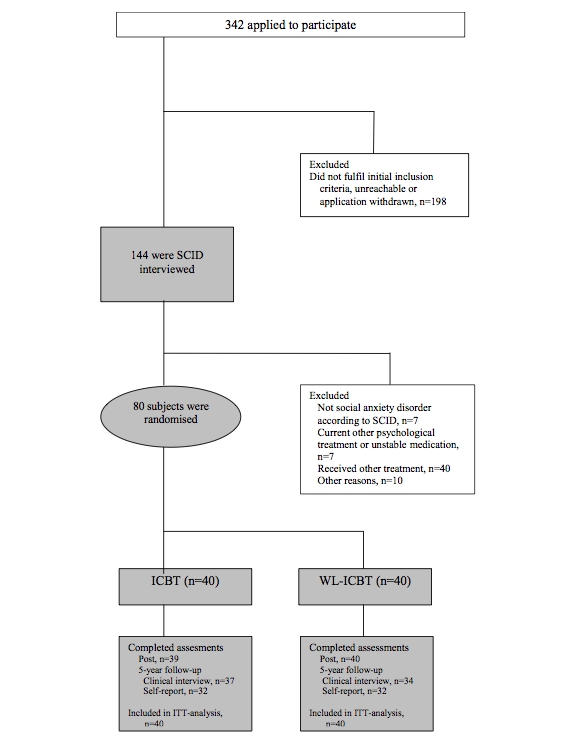
Participant flow

All participants included in the original RCT were eligible to participate in this follow-up study. The main inclusion criteria were the following: participants had to have a primary diagnosis of SAD according to the Structural Clinical Interview for Diagnostic and Statistical Manual of Mental Disorders, 4^th^ edition (DSM-IV) Axis-I Disorders [18]; participants had to agree to undergo no other psychological treatment throughout the original study and keep dosage constant if on prescribed medication for anxiety or depression; and participants had to be at least 18 years old. Main exclusion criteria were not having a computer with Internet access and admitting to another serious disorder (eg, schizophrenia or substance dependence). On average, participants were 35.3 (SD 10.5) years old, and the sample comprised 70% women. Participants in the original RCT were enrolled from January 2005 through March 2005, and recruitment tool place in Uppsala, Sweden. The flow of participants throughout the study is presented in Figure 1. The follow-up study was approved by the regional ethics review board in Stockholm, Sweden, and informed consent was obtained from all participants.

### Outcome Measures

The primary outcome measure was the Liebowitz Social Anxiety Scale-Self-Report (LSAS-SR) [19]. The LSAS-SR measures fear in and avoidance of 24 social situations (13 performance and 11 interaction situations) that are usually difficult for people suffering from SAD. Fear and avoidance in each situation is rated on a 4-point scale from 0 to 3. We also used the Social Interaction Anxiety Scale (SIAS) [20], the Social Phobia Scale (SPS) [20], and the Social Phobia Screening Questionnaire (SPSQ) [1] as complementary measures of social anxiety. The SPS assesses anxiety in 20 performance situations, while the SIAS is constructed to measure anxiety in 20 social interaction situations. Each situation is rated on a 5-point scale ranging from 0 to 5. The SPSQ, designed to screen for SAD using DSMV-IV criteria, was used solely as a dichotomous indicator of SAD diagnosis.

In addition, the Montgomery-Åsberg Depression Rating Scale-Self-report (MADRS-S) [21] and the Beck Anxiety Inventory (BAI) [22] were used as secondary measures to assess depressive symptoms and general anxiety, respectively. MADRS-S comprises 9 items measuring different aspects of depressive symptoms, and each symptom is rated on a 7-point scale. The BAI assesses 21 anxiety symptoms on a 4-point scale from 0 to 3. The Quality of Life Inventory (QOLI) [23] was also administered as a secondary generic outcome measure. The QOLI measures quality of life in16 different domains (eg, work and family). For each domain, the respondent is asked to rate importance on a 3-point scale (from 0 to 2) and the degree of satisfaction on a 6-point scale (from −3 to +3). By multiplying importance by satisfaction, each domain yields a value from −6 to +6.

All measures described above have demonstrated good psychometric properties.

### Clinical Assessment Interview

The SCID-I [18] was used to establish whether participants met diagnostic criteria for SAD at 5-year follow-up. Global improvement was measured by the Clinical Global Impression Improvement Scale (CGI-I) [24]. In addition, information about current and earlier psychological and pharmacological treatments was obtained. Finally, participants were asked to rate to what extent they attributed their improvement/current state to Internet-based CBT.

### Treatment

The Internet-based CBT used in this study has been found efficacious in several randomized controlled trials [4,5,25]. The treatment followed a CBT model that stresses the importance of avoidance and safety behaviors as maintaining factors of SAD [26]. The most central feature of the treatment was a self-help text comprising 9 text modules delivered via the Internet, each covering a specific theme (eg, exposure and cognitive restructuring) including homework exercises.

The introductory module described basic features of SAD and facts about CBT. The topics of modules 2 to 4 were primarily the social anxiety model as presented by Clark and Wells, as well as cognitive restructuring. Modules 5 to 7 introduce safety behavior experiments, exposure exercises, and attention training. Modules 8 and 9 had a main focus on social skills and relapse prevention. The general treatment procedure was that participants read the self-help text, carried out the home work assignments, and reported to their therapist through an online message system.

Throughout the trial, all participants had access to a therapist who supervised the progress and gave feedback on homework exercises. All therapists were clinical psychologists in training during the last semester of their 5-year educational programme. In addition, participants had access to an online discussion forum where they could communicate anonymously with each other. The duration of the treatment was 9 weeks.

### Procedure

The clinical assessment interview was performed by a clinical psychologist with more than 5 years experience in working with structured diagnostic assessments. The interview was conducted by telephone, which has been shown to be a reliable way of assessing psychiatric symptoms [27,28]. The LSAS-SR, SIAS, SPS, SPSQ, MADRS-S, BAI, and QOLI were administered via the Internet, a valid administration format for these instruments [29].

### Statistical Analysis

Statistical analyses were conducted using PASW version 18.0 (SPSS inc, Chicago, IL). While data were analyzed on intent-to-treat basis, we did not apply last observation carried forward (LOCF) to handle missing data as that might have exaggerated the degree to which gains were sustained. Instead, we report the observed means and standard deviations as well as estimated means and standard deviations, as suggested by Gueorguieva and Krystal [30]. Estimated parameters were obtained using a mixed-models approach employing a first order autoregressive covariance structure. The following formula was used for converting standard errors to standard deviations: SD = SE (√n). As all participants received Internet-based CBT, the main analyses entailed no between-group comparisons. However, as half of the sample served as controls in the first phase of the RCT, the two groups are reported separately. We conducted mixed-effect models analysis to assess improvement over time on continuous outcome variables. Nominal data were analyzed with McNemar’s test of change. Effect sizes (Cohen’s *d*) were calculated using the observed means and pooled SDs. 

## Results

### Attrition

Of 80 participants, 71 (89%) underwent the clinical assessment interview and 64 (80%) completed the LSAS-SR, SIAS, SPS, MADRS-S, BAI, and QOLI. There were no statistically significant differences between participants who did not provide follow-up data and those who did regarding gender (c^2^_1_ = 0.39, *P* = .39), age, and social anxiety at baseline or at 1-year follow-up (*t*_1,__67-78_ = 0.40 - 1.74, *P* = .68 - .09). The reasons for not completing the 5-year follow-up are unknown. 

### Social Anxiety Measures

**Figure 2:**
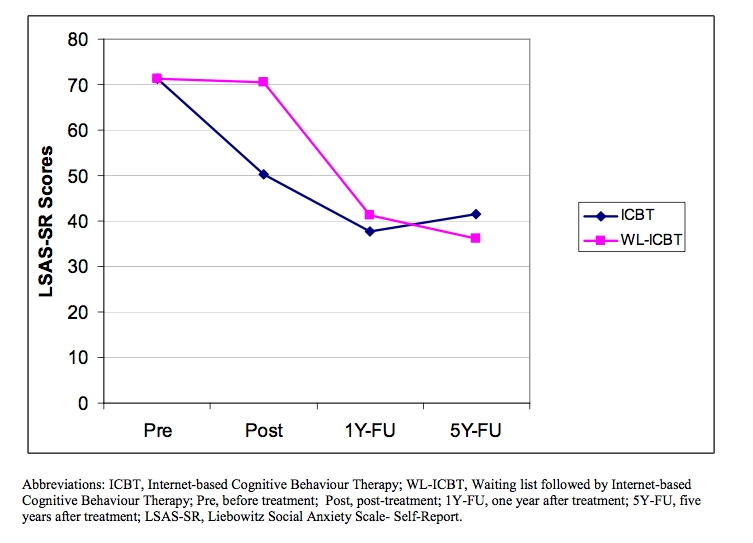
Improvement course on the primary outcome measure LSAS-SR during the follow-up period

The observed and estimated means and SDs as well as effect sizes of the continuous outcome measures are presented in Table 1. Mixed-effect models analysis showed a significant effect of time on the primary outcome measure LSAS-SR, as well as on the SIAS and SPS (*F*_3,__98-102_ = 16.05 - 29.20, *P* < .001). Pairwise comparisons showed that participants in both groups were significantly improved from baseline to 1- and 5-year follow-up on all social anxiety measures (*F*_1,__33-38_ = 15.10 - 90.05, *P* < .001). The CBT group was further improved at 1-year follow-up compared with postassessment, and the WL-CBT group were also improved during this period *(F*_1,__34-35_ = 7.43 - 40.42, *P* = .01 - .001). There were no significant changes on the LSAS and SPS between 1- and 5-year follow-up (*F*_1,28,__32_ = 0.22, 0.93, *P* = .64 - .13). In the WL-CBT group but not in the CBT group, participants were further improved on the SIAS at 5-year follow-up compared with 1-year follow-up (*F*_1,29_ = 7.85 *P* = .01). Figure 2 displays changes on the primary outcome measure LSAS-SR across assessment points. Note that as we used LOCF to handle missing data in the original article, there are minimal and nonsignificant discrepancies in the present report compared with the original regarding parameters at postassessment and 1-year follow-up.

**Table 1:** Observed and estimated means, SDs, and effect sizes (Cohen’s *d*) on continuous outcome measures

Measure and Group n = 40 (CBT and WL-CBT)		Pre M (SD)	Post M (SD)	1-Year Follow-up M (SD)	Observed 5-year Follow-up M (SD)	Estimated 5-Year Follow-up M (SD)	Effect Size Within Pre 1-Year Follow-up (95%CI)	Effect Size Within Pre 5-Year Follow-up (95%CI)
**LSAS-SR**								
	CBT	71.3 (22.5)	50.3 (21.0)	37.7 (17.7)	41.5 (23.7)	41.6 (20.9)	1.65 (1.11–2.15)	1.30 (0.77–1.79)
	WL-CBT	71.3 (24.9)	70.4 (27.6)	41.3 (29.0)	36.3 (25.3)	38.9 (24.9)	1.12 (0.61-1.60)	1.40 (0.86-1.90)
**SIAS**								
	CBT	51.0 (14.2)	38.5 (13.9)	32.8 (14.9)	36.3 (16.8)	36.1 (14.7)	1.25 (0.73-1.75)	0.95 (0.45-1.43)
	WL-CBT	46.5 (17.9)	46.4 (18.7)	31.7 (18.3)	24.6 (14.7)	25.9 (15.8)	0.81 (0.34-1.27)	1.32 (0.79-1.82)
**SPS**								
	CBT	39.2 (15.3)	25.2 (12.0)	19.0 (12.0)	22.6 (18.4)	22.6 (14.7)	1.46 (0.94-1.95)	0.98 (0.48-1.47)
	WL-CBT	36.4 (17.1)	35.7 (16.4)	20.0 (14.7)	16.6 (16.4)	17.5 (15.3)	1.02 (0.53-1.49)	1.18 (0.66-1.67)
**MADRS-S**								
	CBT	14.9 (7.8)	10.4 (6.3)	9.7 (7.0)	9.6 (7.8)	10.5 (7.9)	0.70 (0.23-1.16)	0.68 (0.20-1.15)
	WL-CBT	15.7 (9.3)	16.3 (10.2)	10.9 (8.5)	7.7 (8.9)	8.3 (9.0)	0.54 (0.08-0.99)	0.88 (0.39-1.36)
**BAI**								
	CBT	16.1 (7.4)	9.8 (5.8)	10.4 (7.2)	10.6 (10.4)	10.5 (7.9)	0.87 (0.39-1.33)	0.63 (0.15-1.10)
	WL-CBT	16.2 (9.6)	15.3 (9.4)	11.8 (9.2)	8.3 (9.8)	8.7 (9.0)	0.68 (0.21-1.14)	0.81 (9.32-1.28)
**QOLI**								
	CBT	0.8 (1.9)	1.3 (2.0)	1.7 (1.5)	1.9 (1.7)	1.7 (1.7)	0.55 (0.09-1.00)	0.63 (0.15-1.10)
	WL-CBT	0.6 (1.9)	0.4 (1.6)	1.4 (1.8)	2.1 (1.8)	1.9 (1.7)	0.41 (−0.06 to 0.86)	0.77 (0.28-1.25)

### Depressive Symptoms, General Anxiety, and Quality of Life

Effect sizes and observed and estimated parameters of secondary outcome measures are presented in Table 1. Mixed-effect models analysis showed a significant effect of time on the MADRS-S, BAI, and QOLI (*F*_3,__97-104_ = 4.64 - 9.78, *P* = .01 - .001). Pairwise comparisons showed that participants in both groups were significantly improved from baseline to 1- and 5-year follow-up on MADRS-S, BAI, and QOLI (*F*_1,32-40_ = 4.7 - 30, *P* = .04 - .001). The WL-CBT was improved at 1-year follow-up compared with postassessment on these measures (*F*_1,34,35_ = 12.12 - 13.83, *P* < .001), whereas the CBT group was not (*F*_1,35-37_ = 0.36 - 3.09, *P* = .55 - .09). There were no changes on these measures from 1- to 5-year follow-up (*F*_1,28,33_ = 0.01 - 3.80, *P* = .94 - .06). 

### Clinical Assessment Interview

#### Global Improvement and Diagnostic Assessment

**Figure 3:**
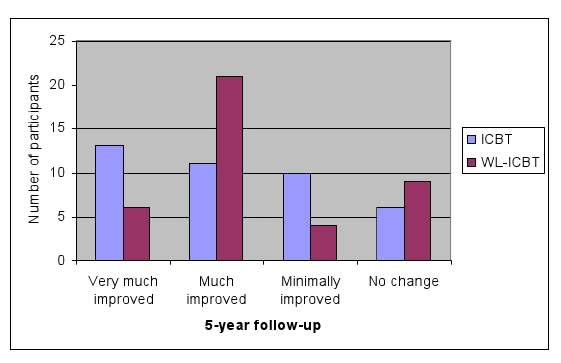
Clinical Global Impression Improvement (CGI-I) scores at 5-year follow-up (dropouts are considered non-responders)

Figure 3 displays CGI-I scores at 5-year follow-up for both groups. At this time, 60% of participants (24/40) in the CBT group and 67.5% (27/40) in the WL-CBT group were considered very much or much improved, that is, responders. At 5-year follow-up, 48% of participants (19/40) in both groups no longer met diagnostic criteria for SAD according to the clinician assessment (counting dropouts as nonresponders). McNemar’s test showed that this was a statistically significant change compared with baseline (*P* < .001). According to the SPSQ, 40% (16/40) of the participants in the CBT group and 45% (18/40 in the WL-CBT group no longer met criteria for SAD (counting dropouts as nonresponders).

#### Participants’ Attribution of Improvement

Participants were asked to rate to what extent they attributed their improvement to the Internet-based CBT on a Likert-scale from 0 to 100 (0 = any improvement is completely unrelated to Internet-based CBT, 50 = any improvement is equally due to Internet-based CBT and other causes, and 100 = any improvement is completely due to Internet-based CBT). In the CBT group, the average score was 60.3 (SD 26.9) and the corresponding WL-CBT score was 61.8 (SD 25.9).

#### Other Psychological and Psychotropic Treatments Received Since Internet-based CBT

At 5-year follow-up, 10% (4/40) participants in the CBT group had received some form of psychological treatment (all reasons included) after Internet-based CBT. This was 11% (4/37) if counting completers only, that is, those who provided data. The corresponding percent in the WL-CBT + WL group was 17.5% (7/40). This was 21% (7/34) if counting completers only. In the CBT group, 1 of the 40 participants (2.5%), or 1 of 37 (2.7%) if counting completers only, was taking psychotropic medication, that is, selective serotonin reuptake inhibitors (SSRIs) at the time of the 5-year follow-up assessment, although 4 of 40 participants (10%), or 4 of 37 (11%) if counting completers only, had started and discontinued psychotropic medication at some point during the follow-up period (all SSRIs). In the WL-CBT group, the corresponding numbers were 3 of 40 (7.5%), or 3 of 37 (8%) if counting completers only, and 5 of 40 (12.5%), or 5 of 34 (15%) if counting completers only, respectively (all had been taking SSRIs). The status of the 11% (9/80) dropouts regarding medication is unknown.

## Discussion

### Main Findings

The aim of this study was to evaluate the 5-year effect of Internet-based CBT for SAD by assessing participants who had received Internet-based CBT within the context of an RCT. The results showed that improvements on measures of social anxiety at 1-year follow-up were sustained 5 years after treatment. Overall, effect sizes were large on measures of social anxiety. In addition, improvements regarding depressive symptoms, general anxiety, and quality of life were also sustained at 5-year follow-up. The results of this study indicate that participants receiving Internet-based CBT for SAD are moderately improved immediately following treatment but make further improvements within the following year. Improvements made at 1-year follow-up are, in turn, long-term enduring.

The effect sizes in this study are in line with those reported in studies investigating the long-term effects of conventional CBT for SAD [13,31]. They are also in line with results from a previous independent 2.5-year follow-up study of Internet-based CBT for SAD [16]. The major strength of this study is that attrition rates were low making the generalizability of the findings high. The low attrition rates were also reflected in the small differences in the observed and estimated estimates. Furthermore, participants attributed their improvement to Internet-based CBT to a large extent, and few had commenced other forms of psychological or psychotropic treatments after completing Internet-based CBT. Taken together, this suggests that the reduction of social anxiety observed at 5-year follow-up was largely an effect of Internet-based CBT.

### Clinical Implications

There are several clinical implications of our findings. First, if Internet-based CBT for SAD has sustained effects over longer time periods, it is highly likely that it is a cost-effective treatment. We did not collect economic data in this study; however, results of a study by Titov and coworkers have demonstrated that Internet-based CBT is likely more cost-effective than group CBT due to lower costs of treatment [32]. Second, it may also be that Internet-based CBT confers benefits in another way compared with conventional therapies, since the material can be saved and used as reminders long after the treatment has ended. The effect sizes found in the present study, which are in parity with those found in trials investigating conventional CBT, suggest that Internet-based CBT has some qualities that compensate for the lack of face-to-face contact. Intriguingly, in the original trial [6], a basic patient satisfaction rating showed that 94% of the participants were satisfied with the treatment and that 91% of the participants found the feedback from the therapists to be good or excellent. This suggests that it is possible to have a good therapeutic relationship online, which has also been reported in other studies on Internet-based CBT [33].

Third, Internet-based CBT may in the future be used as a complement to conventional CBT and pharmacotherapy, as it probably can be combined with these two treatments. Internet-based CBT might enable more efficient use of health care resources, that is, as Internet-based CBT requires less therapist time, more resources can be made available for patients who need a more intensified treatment. This, in turn, could lead to a larger total proportion of treatment responders. Future research should more clearly link symptom improvement to the treatment provided and the extent to which strategies learned in treatment are used to prevent recurrence.

### Limitations

The present study has several limitations, and we view the following as most important. First, common to most long-term follow-up trials, there was no randomization to a control condition with which treatment results could be compared at 5-year follow-up. However, considering the chronicity of SAD [3], we find it unlikely that improvements are due to spontaneous recovery. Furthermore, it is improbable that nonspecific treatment effects such as attention from a therapist would generate improvements that are enduring over 5 years. Second, we did not use a behavioral test to assess social anxiety, which would have been a more objective measure than the ones used. For example, Heimberg and coworkers used a test where participants were exposed to personally tailored social situations while using heart rate monitoring equipment to assess bodily symptoms of anxiety [34]. Nonetheless, we view the combination of clinician assessment and administration of questionnaires with good psychometric properties as a valid assessment method. Third, the intervals between the follow-ups were not regular, and it is not possible to infer symptom levels between the follow-ups (eg, 3 years posttreatment). As clinical assessment interviews were only conducted at pretreatment and 5-year follow-up, this uncertainty also applies to diagnostic status. However, as symptoms of SAD are not known to fluctuate spontaneously, we find it unlikely that levels of social anxiety in the present sample varied greatly between 1- and 5-year follow-up. Finally, although attrition rates were low, 11% of the participants did not attend the assessment interview. Of course, it might be that these individuals are less improved than those who participated in the 5-year follow-up assessment. However, even if those participants were nonresponders, it would have only a marginal effect on the effect size estimates. We also view the types of analyses performed, where models were created using all available data, yielded the best estimate, as last observation carried forward could have overestimated the long-term effect.

In spite of these limitations, we regard the results of the present study as important as they are the first to demonstrate that Internet-based CBT for SAD can yield large effects that are enduring over 5 years. 

## Abbreviations

BAI: Beck Anxiety Inventory
CBT: cognitive behavior therapy
CGI-I: Clinical Global Impression Improvement Scale
CI: confidence interval
DSMV-IV: Diagnostic and Statistical Manual of Mental Disorders, 4th edition
LOCF: last observation carried forward
LSAS-SR: Liebowitz Social Anxiety Scale-Self-Report
MADRS-S: Montgomery-Åsberg Depression Rating Scale-Self-Report
QOLI: Quality of Life Inventory ()
RCT: randomized controlled trial
SAD: social anxiety disorder
SIAS: Social Interaction Anxiety Scale
SPS: Social Phobia Scale
SPSQ: Social Phobia Screening Questionnaire
SSRI: selective serotonin reuptake inhibitor
